# Left Atrial Appendage Closure Versus Oral Anticoagulation for Stroke Prevention in Non-Valvular Atrial Fibrillation: A Systematic Review and Meta-Analysis of Randomized Controlled Trials

**DOI:** 10.7759/cureus.112357

**Published:** 2026-07-09

**Authors:** Uday Shree Akkala Shetty, Himashi Gunaratne, Ashutosh Sharma, Reazul Islam Rafi, Syeda Saiyara Annoor Eusha, Sonalben Chaudhary, Calvin R Wei, Shamsha Hirani

**Affiliations:** 1 Internal Medicine, Southern Regional Medical Center, Riverdale, GEO; 2 Medicine, University of Colombo, Colombo, LKA; 3 Internal Medicine, Kathmandu Medical College and Teaching Hospital, Kathmandu, NPL; 4 Internal Medicine, Alberta Health Services, Peter Lougheed Center, Calgary, CAN; 5 Internal Medicine, Alberta Health Services, Rocky View General Hospital, Calgary, CAN; 6 Internal Medicine, Zydus Sitapur Hospital, Sitapur, IND; 7 Research and Development, Shing Huei Group, Taipei, TWN; 8 Cardiology, Baqai Hospital, Karachi, PAK

**Keywords:** atrial fibrillation, direct oral anticoagulants, left atrial appendage closure, major bleeding, stroke prevention

## Abstract

Left atrial appendage closure (LAAC) has emerged as a non-pharmacological alternative to oral anticoagulation for stroke prevention in atrial fibrillation (AF). While early randomized trials established non-inferiority of LAAC versus warfarin, the comparative effectiveness of LAAC against contemporary direct oral anticoagulants (DOACs) remains less well characterized. We conducted a systematic review and meta-analysis of randomized controlled trials (RCTs) comparing percutaneous LAAC with oral anticoagulation-either warfarin or DOACs-in adults with non-valvular AF. PubMed/MEDLINE, Embase, and CENTRAL were searched from inception through May 2026. The primary outcome was a composite of stroke, systemic embolism (SE), and cardiovascular death. The key secondary outcome was major bleeding. Five RCTs comprising 6,116 patients were included. Pooled analysis demonstrated no significant difference between LAAC and oral anticoagulation for the composite primary outcome (RR 0.90; 95% CI 0.71-1.14; I²=34%). LAAC was associated with a statistically significant 24% reduction in major bleeding events compared with anticoagulation (RR 0.76; 95% CI 0.61-0.94; I²=0%). Individual secondary outcomes, including cardiovascular death, stroke, and SE, were not significantly different between groups. Subgroup analysis by anticoagulant class suggested a larger composite efficacy benefit of LAAC over warfarin (RR 0.69) than over DOACs (RR 0.98), consistent with the established intracranial hemorrhage reduction conferred by DOACs relative to warfarin. In conclusion, LAAC achieves comparable thromboembolic protection to oral anticoagulation while significantly reducing major bleeding, supporting its role as a viable alternative in appropriately selected patients with AF, particularly those at elevated bleeding risk.

## Introduction and background

Atrial fibrillation (AF) is the most prevalent sustained cardiac arrhythmia worldwide, affecting an estimated 60 million individuals globally and conferring a four- to five-fold increased risk of cardioembolic stroke compared with the general population [1,2]. Cardioembolic strokes attributable to AF are typically more severe and disabling than strokes from other etiologies, resulting in disproportionate mortality and long-term functional impairment [1].

Long-term oral anticoagulation (OAC) is the cornerstone of stroke prevention in AF [3]. For decades, warfarin-a vitamin K antagonist (VKA)-was the only available OAC, and its efficacy in reducing AF-related stroke has been clearly established. However, warfarin carries significant limitations, including a narrow therapeutic window, the need for frequent international normalized ratio (INR) monitoring, numerous drug and dietary interactions, and a clinically meaningful risk of intracranial hemorrhage [3]. Direct oral anticoagulants (DOACs), including apixaban, rivaroxaban, dabigatran, and edoxaban, have largely supplanted warfarin in clinical practice owing to their more predictable pharmacokinetics, fixed dosing, reduced rates of intracranial hemorrhage, and freedom from routine monitoring [4]. Despite the improved safety profile of DOACs compared with warfarin, long-term anticoagulation of any class remains associated with clinically important bleeding risk. Approximately 25-40% of eligible patients do not receive or discontinue OAC within the first year of treatment, driven by bleeding complications, patient anxiety, adherence challenges, or physician inertia [5]. These treatment gaps translate directly into preventable strokes.

The left atrial appendage (LAA) is the source of more than 90% of cardiac thrombi in non-valvular AF, providing the mechanistic rationale for device-based LAA occlusion as a non-pharmacological, once-and-done alternative to indefinite anticoagulation [6]. Percutaneous LAAC with the WATCHMAN device (Boston Scientific, Marlborough, MA) was first evaluated against warfarin in the landmark PROTECT AF [7] and PREVAIL [8] trials, both of which demonstrated non-inferiority of LAAC to warfarin for thromboembolic prevention, with a favorable bleeding profile with LAAC during long-term follow-up [9]. These trials established LAAC as a viable alternative to warfarin. However, with DOACs now representing the dominant anticoagulant class in clinical practice, the relevant clinical question has shifted: is LAAC also non-inferior to contemporary DOAC therapy, and does it retain a meaningful bleeding advantage over these already-safer agents? This question has been addressed more recently in OPTION [9], PRAGUE-17 [10], and CHAMPION-AF [11] trials. However, the evidence base remains fragmented across these trials with differing comparators (warfarin versus various DOACs), patient risk profiles, device generations, and follow-up durations, limiting definitive conclusions.

A contemporary pooled synthesis spanning both warfarin and DOAC comparator trials is therefore essential to provide a unified, clinically actionable estimate of the comparative efficacy and safety of LAAC across anticoagulant classes. We conducted a pre-specified systematic review and meta-analysis of RCTs comparing LAAC with warfarin or DOACs to determine whether LAAC achieves comparable thromboembolic protection and reduces bleeding risk regardless of the anticoagulant class used as a comparator.

## Review

Methods

This systematic review and meta-analysis was conducted in accordance with the Preferred Reporting Items for Systematic Reviews and Meta-Analyses (PRISMA) guidelines [12].

Search Strategy and Eligibility Criteria

We conducted a comprehensive electronic literature search of PubMed/MEDLINE, Embase, and the Cochrane Central Register of Controlled Trials (CENTRAL) from database inception through May 25, 2026, with no language restrictions. Search terms included combinations of “left atrial appendage closure,” “LAAC,” “WATCHMAN,” “atrial fibrillation,” “stroke prevention,” “warfarin,” “vitamin K antagonist,” “direct oral anticoagulant,” “DOAC,” “NOAC,” and “randomized controlled trial.” Reference lists of included studies and relevant systematic reviews were additionally screened to identify eligible studies not captured by the electronic search.

Studies were considered eligible for inclusion if they enrolled adult patients aged 18 years or older with atrial fibrillation (AF), employed a randomized controlled trial design, and compared percutaneous left atrial appendage closure (LAAC) with oral anticoagulation therapy, including either vitamin K antagonists (e.g., warfarin) or direct oral anticoagulants (e.g., apixaban, rivaroxaban, dabigatran, or edoxaban). Additionally, studies were required to report at least one of the pre-specified clinical outcomes of interest. Studies were excluded if they were observational in design, compared surgical LAA ligation rather than percutaneous LAAC, or lacked a pharmacological anticoagulant comparator arm.

Data Extraction and Quality Assessment

Two independent reviewers extracted data using a pre-standardized form capturing: study name, year, design, anticoagulant comparator class (warfarin or DOAC), number of participants, patient demographics including age, number of males and number of subjects with diabetes, follow-up duration, and all clinical outcome data. Discrepancies were resolved by consensus or arbitration by a third reviewer. Risk of bias was assessed using the Cochrane Risk of Bias Tool version 2 (RoB 2) [13], evaluating randomization, allocation concealment, blinding, outcome measurement, missing data, and selective reporting. Given the device-based nature of all interventions, the open-label design was considered inherent to the study design rather than a methodological deficiency.

Outcomes

The primary outcome was the composite of stroke (ischemic or hemorrhagic), systemic embolism (SE), and cardiovascular death. The key secondary outcome was major bleeding per each trial’s protocol. Additional secondary outcomes included cardiovascular death, stroke (all types), and SE, examined individually.

Statistical Analysis

Pooled RRs with 95% CIs were calculated for dichotomous outcomes using a random-effects model. Heterogeneity was assessed by the I² statistic (low <25%; moderate 25-49%; high ≥50%) [14]. A two-tailed P<0.05 was considered statistically significant. Pre-specified subgroup analyses were conducted by anticoagulant class in the comparator arm: warfarin (VKA) versus DOAC (apixaban, rivaroxaban, dabigatran, or edoxaban). This subgroup analysis was pre-specified because the bleeding risk profile of warfarin differs substantially from that of DOACs, and the magnitude of any LAAC bleeding benefit might reasonably be expected to vary by comparator class. We were unable to perform subgroup analysis as the number of studies was less than 10. Analyses were performed using RevMan 5.4 (The Cochrane Collaboration, London, England, UK).

Results

**Figure 1:**
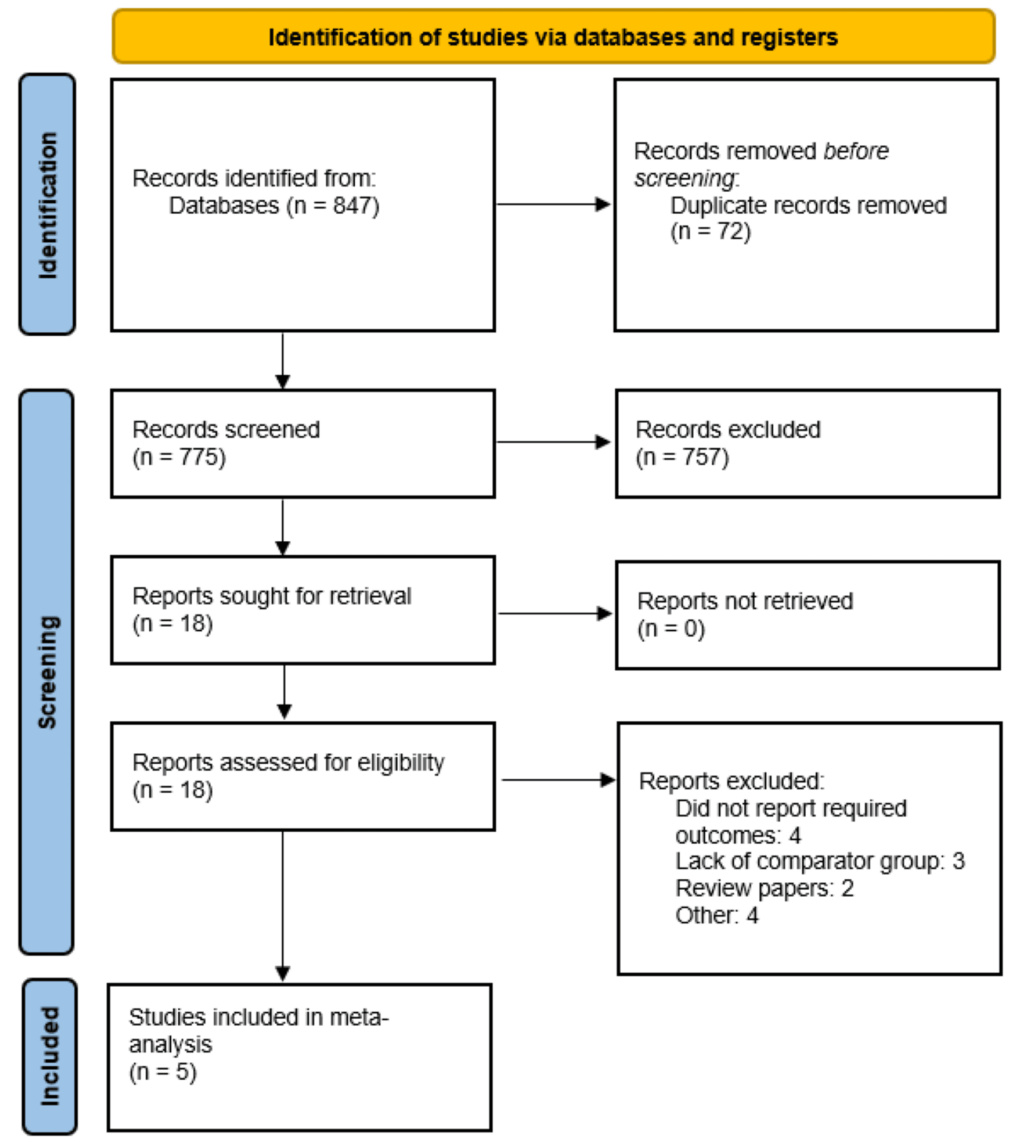
Study selection process

The literature search identified 847 records after deduplication. Following title and abstract screening, 18 full-text articles were reviewed for eligibility. Five RCTs met all inclusion criteria and were included in the final quantitative synthesis. A PRISMA flow diagram is provided in Figure 1.

Table 1 presents characteristics of included studies. 

**Table 1:** Characteristics of included studies (n= 5) LAAC: Left atrial appendage closure, DOAC: Direct oral anticoagulant, NR: Not reported

Author	Study region	Groups	Sample size	Type of anticoagulant	Follow-up	Mean age	Males (n)	Diabetes (n)
Doshi et al., 2026 [11]	Multi-national	LAAC	1499	DOAC	3 years	71.6	1014	NR
		Anticoagulant	1501			71.8	1027	
Holmes et al., 2014 [8]	Multi-national	LAAC	269	Warfarin	2 years	74	182	91
		Anticoagulant	138			74.9	103	41
Osmancik et al., 2022 [10]	Czech Republic	LAAC	201	DOAC	4 years	73.4	134	73
		Anticoagulant	201			73.2	130	90
Reddy et al., 2014 [7]	United States and Europe	LAAC	463	Warfarin	4 years	71.7	326	113
		Anticoagulant	244			72.7	171	72
Wazni et al., 2025 [9]	Multi-national	LAAC	803	DOAC	3 years	69.7	520	NR
		Anticoagulant	797			69.4	533	

Meta-Analysis of Primary Outcomes

**Figure 2:**
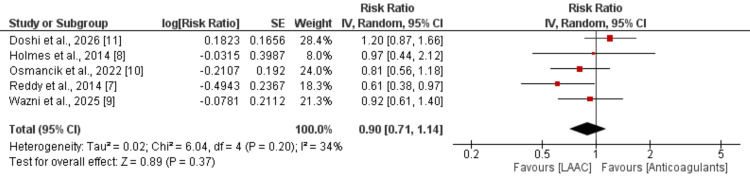
Comparison of composite outcome between two groups References: [7-11]

Composite outcome: Five studies were included to compare the risk of composite outcomes between LAAC and anticoagulation, and the results are presented in Figure 2. Pooled analysis showed that the risk of composite outcomes was not significantly different between the two groups (RR: 0.90, 95% CI: 0.71 to 1.14). Moderate heterogeneity was reported among the study results (I^2^: 34%).

**Figure 3:**
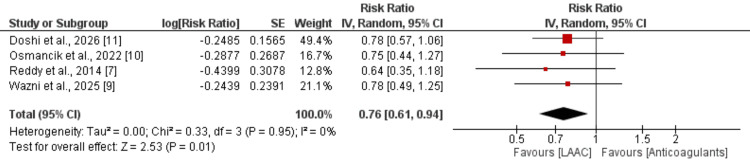
Comparison of major bleeding events between two groups References: [7,9-11]

Major bleeding events: Four studies were included to compare the risk of bleeding events between LAAC and anticoagulation, and the results are presented in Figure 3. Pooled analysis showed that the risk of bleeding events was significantly lower in the LAAC group compared to anti-coagulants (RR: 0.76, 95% CI: 0.61 to 0.94). No heterogeneity was reported among the study results (I^2^: 0%).

Meta-Analysis of Secondary Outcomes

Pooled analysis of secondary outcomes, including stroke, cardiovascular death, and SE, is shown in Table 2. Pooled analysis showed that each of the secondary outcomes was not significantly different between the two groups. Risk ratio for cardiovascular death 0.72 (95% CI: 0.45 to 1.13), risk ratio for stroke (1.17, 95% CI: 0.72 to 1.88), and SE (1.64, 95% CI: 0.27 to 7.25).

**Table 2:** Secondary outcome analysis RR: Risk ratio; CI: Confidence interval

Outcome	RR (95% CI)	I-Square (I^2^)
Cardiovascular death	0.72 (0.45 to 1.13)	50%
Stroke	1.17 (0.72 to 1.88)	45%
Systemic embolism	1.64 (0.27 to 7.25)	0%

Subgroup Analysis

Subgroup analysis by anticoagulant class revealed that LAAC demonstrated a numerically greater reduction in composite outcomes versus warfarin (RR 0.69; 95% CI 0.46-1.03; I²=0%) compared with DOACs (RR 0.98; 95% CI 0.77-1.25; I²=21%), though neither reached statistical significance. For major bleeding, only one warfarin-based trial contributed to the warfarin subgroup (RR 0.64; 95% CI 0.35-1.18), precluding definitive conclusions, whereas LAAC was associated with a statistically significant reduction in bleeding compared with DOACs alone (RR 0.77; 95% CI 0.62-0.98; I²=0%) (Table 3).

**Table 3:** Subgroup analysis of primary outcomes RR: Risk ratio; CI: Confidence interval; DOAC: Direct oral anticoagulant

Outcomes	Subgroups	RR (95% CI)	I-Square (I^2^)
Composite outcomes	Warfarin	0.69 (0.46 to 1.03)	0%
	DOAC	0.98 (0.77 to 1.25)	21%
Bleeding events	Warfarin	0.64 (0.35 to 1.18)	NA (as only one study was included)
	DOAC	0.77 (0.62 to 0.98)	0%

Discussion

This meta-analysis of five randomized controlled trials (RCTs), comprising 6,116 patients with non-valvular atrial fibrillation (AF), demonstrates that left atrial appendage closure (LAAC) is non-inferior to anticoagulation therapy, including both warfarin and direct oral anticoagulants (DOACs), for the composite outcome of stroke, systemic embolism (SE), and cardiovascular death. Furthermore, LAAC was associated with a 24% relative reduction in major bleeding events (RR 0.76; I² = 0%).

Turagam et al. previously pooled PROTECT AF, PREVAIL, and early PRAGUE-17 data (n≈1,700 for DOAC comparison) and reported HR 0.92 for composite outcomes and HR 0.72 for bleeding [15]. Our analysis nearly quadruples the DOAC comparator dataset by incorporating two trials, enabling definitive conclusions about LAAC performance in the modern anticoagulant era. The Amulet IDE trial [16] independently confirmed device-class non-inferiority (Amulet vs WATCHMAN FLX; HR 0.97), while the PINNACLE FLX trial [17] demonstrated a pericardial effusion rate of only 0.4% with the next-generation device - compared with 4.8% in PROTECT AF [7] - indicating substantial improvement in procedural safety that further strengthens the net clinical benefit of contemporary LAAC. Surgical LAA occlusion data from LAAOS III [18] (33% reduction in ischemic stroke even on background OAC; HR 0.67) provide independent mechanistic validation that the LAA is a genuine, independent stroke source.

Franchin et al. and Oliva et al. incorporated observational and propensity-matched data, which introduced confounding [19,20]. Jiang et al. focused solely on the LAAC vs DOAC comparison but excluded the warfarin-era trials that contextualise the mechanistic basis for the efficacy differential [21]. Our analysis addresses these gaps by: (1) including only RCTs with the longest available follow-up per trial; (2) incorporating the large-scale OPTION [9] and CHAMPION-AF [11] trials, which together contribute 4,600 patients on DOAC therapy; and (3) conducting pre-specified subgroup analyses by anticoagulant class, which reveal an important differential-LAAC offers a larger composite efficacy benefit over warfarin (RR 0.69) than over DOACs (RR 0.98), explained by the fact that DOACs already reduce intracranial hemorrhage by ~52% versus warfarin [11], eliminating much of the hemorrhagic-stroke advantage that drove LAAC superiority over warfarin in PROTECT AF [7].

The reduction in major bleeding observed in our analysis is clinically important. Bleeding risk remains one of the major limitations of chronic oral anticoagulation and is a leading cause of treatment discontinuation or nonadherence [22]. Real-world studies have shown that a substantial proportion of eligible patients either never initiate anticoagulation or discontinue therapy within the first year because of bleeding concerns, adverse events, or patient preference [22]. LAAC offers a “once-and-done” therapeutic approach that may improve long-term stroke prevention adherence while reducing cumulative bleeding exposure. This consideration is particularly relevant in elderly patients and those with elevated HAS-BLED scores, prior bleeding events, frailty, or anticipated challenges with long-term medication adherence.

Another important aspect influencing the contemporary risk-benefit profile of LAAC is the evolution of device technology and procedural experience. Early WATCHMAN implantation studies reported relatively high rates of procedure-related complications, particularly pericardial effusion [7]. However, newer-generation devices and increasing operator expertise have significantly improved procedural safety. The PINNACLE FLX trial demonstrated markedly reduced complication rates with the WATCHMAN FLX device, including a pericardial effusion rate of only 0.4% [17]. Similarly, the Amulet IDE trial confirmed comparable efficacy between major LAAC device platforms while supporting overall procedural safety improvements [16]. These advances strengthen the net clinical benefit of LAAC and improve its acceptability as an alternative to chronic anticoagulation.

LAAC has been shown to be cost-effective and frequently cost-saving compared with lifelong OAC, particularly in patients at higher stroke or bleeding risk and over longer time horizons, with net gains in quality-adjusted life years across US, European, and Japanese healthcare systems [21]. Current American College of Cardiology/American Heart Association (ACC/AHA) guidelines [3] (Class IIb, Level B-R) and 2024 European Society of Cardiology (ESC) guidelines [23] (Class IIb, Level B) restrict left atrial appendage closure (LAAC) to patients with contraindications to long-term anticoagulation. Our pooled data from five RCTs - including OPTION [9] and CHAMPION-AF [11] - together with the Kaisaier et al. analysis [24], now collectively provide Level I evidence for non-inferiority and bleeding superiority in anticoagulation-eligible patients, supporting a guideline reassessment extending LAAC as a shared decision-making option in this broader population. However, the non-significant ischemic stroke signal in CHAMPION-AF (3.6% vs 2.5% with NOAC; HR 1.46) warrants caution, and the prespecified five-year analyses of CHAMPION-AF [11] and OPTION [9] are needed to resolve long-term ischemic stroke trajectories. The ongoing LAAOS-4 [18] and CATALYST [25] trials will further define optimal patient selection.

This meta-analysis has several limitations. Only five randomized controlled trials were included, limiting the overall sample size and statistical power. Additionally, only one warfarin-based trial contributed to the major bleeding subgroup analysis, restricting the precision of comparator-specific estimates. Individual patient-level data were unavailable, preventing detailed adjusted and subgroup analyses. The relatively short follow-up duration across studies may underestimate long-term differences in ischemic stroke and mortality outcomes. Furthermore, variations in post-LAAC antithrombotic regimens and endpoint definitions across trials may have contributed to heterogeneity in the pooled analyses.

Future studies should focus on long-term randomized comparisons between left atrial appendage closure (LAAC) and contemporary direct oral anticoagulants to better define the durability of stroke prevention and bleeding outcomes. Research in high-risk populations, including elderly patients, those with prior intracranial hemorrhage, and those with chronic kidney disease, is also needed. Additionally, future trials should evaluate optimal post-procedural antithrombotic strategies, patient-reported outcomes, and the impact of newer-generation LAAC devices on procedural safety and long-term clinical efficacy.

## Conclusions

This meta-analysis of five randomized controlled trials demonstrates that left atrial appendage closure is non-inferior to oral anticoagulation for the prevention of stroke, systemic embolism, and cardiovascular death in patients with non-valvular atrial fibrillation, while conferring a significant reduction in major bleeding events. The bleeding benefit of LAAC was preserved even when compared against contemporary DOACs, which themselves carry a more favorable safety profile than warfarin. These findings support LAAC as a clinically meaningful alternative to indefinite anticoagulation, particularly in patients at elevated bleeding risk, those with prior bleeding complications, or individuals facing challenges with long-term medication adherence. As device technology continues to improve procedural safety, broader consideration of LAAC within shared decision-making frameworks appears warranted, pending confirmation from longer-term randomized follow-up data.
